# Insight into the Role of Extracellular Vesicles in Lysosomal Storage Disorders

**DOI:** 10.3390/genes10070510

**Published:** 2019-07-06

**Authors:** Brunella Tancini, Sandra Buratta, Krizia Sagini, Eva Costanzi, Federica Delo, Lorena Urbanelli, Carla Emiliani

**Affiliations:** 1 Department of Chemistry, Biology and Biotechnology, University of Perugia, Via del Giochetto, 06123 Perugia, Italy; 2 Centro di Eccellenza sui Materiali Innovativi Nanostrutturati (CEMIN), University of Perugia, Via del Giochetto, 06123 Perugia, Italy

**Keywords:** extracellular vesicles, lysosomal storage disorders, exosomes, phospholipidosis, autophagy, lysosomal dysfunction

## Abstract

Extracellular vesicles (EVs) have received increasing attention over the last two decades. Initially, they were considered as just a garbage disposal tool; however, it has progressively become clear that their protein, nucleic acid (namely miRNA and mRNA), and lipid contents have signaling functions. Besides, it has been established that cells release different types of vesicular structures for which characterization is still in its infancy. Many stress conditions, such as hypoxia, senescence, and oncogene activation have been associated with the release of higher levels of EVs. Further, evidence has shown that autophagic–lysosomal pathway abnormalities also affect EV release. In fact, in neurodegenerative diseases characterized by the accumulation of toxic proteins, although it has not become clear to what extent the intracellular storage of undigested materials itself has beneficial/adverse effects, these proteins have also been shown to be released extracellularly via EVs. Lysosomal storage disorders (LSDs) are characterized by accumulation of undigested substrates within the endosomal–lysosomal system, due either to genetic mutations in lysosomal proteins or to treatment with pharmacological agents. Here, we review studies investigating the role of lysosomal and autophagic dysfunction on the release of EVs, with a focus on studies exploring the release of EVs in LSD models of both genetic and pharmacological origin. A better knowledge of EV-releasing pathways activated in lysosomal stress conditions will provide information on the role of EVs in both alleviating intracellular storage of undigested materials and spreading the pathology to the neighboring tissue.

## 1. Introduction

**Figure 1:**
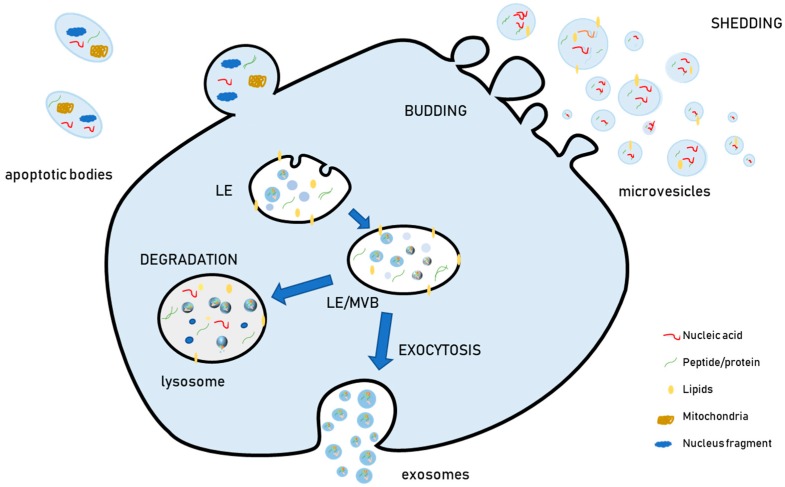
Schematic representation of extracellular vesicle (EV) biogenesis and release. The late endosomes (LEs) become full of small (30–120 nm) ILVs (Intraluminal Vesicles) and take the name of MVBs (Multivesicular Bodies), which can either fuse with lysosomes to degrade their cargo, or with the plasma membrane to release their cargo extracellularly (exosomes). Microvesicles originate from the outward budding of the plasma membrane and, as soon as they are produced, they are released extracellularly. Note that microvesicles are more heterogeneous in size (100–1000 nm). Apoptotic bodies are a heterogenous population of vesicles (50–500 nm) released from cells undergoing apoptosis.

The term “extracellular vesicles” (EVs) has been proposed to designate the membrane-surrounded particles that are released outside cells and are retrieved in vitro in cell culture media and in vivo in all investigated biological fluids. For instance, they have been isolated from blood, urine, saliva, milk, tumor exudates, cerebrospinal fluid, and amniotic liquid [1,2]. During the last decade, evidence has shown that EVs have heterogeneous biophysical features (size, density), biochemical content (proteins, lipids, nucleic acids), and cellular biogenesis [3,4,5,6]. Due to this heterogeneity, EVs have been grouped into different categories and currently, the most widely used EV classification relies on their biogenesis. According to this aspect, EVs are classified as microvesicles or ectosomes (generated by outward budding from the plasma membrane), exosomes (generated by the inward budding of the late endosomal membrane, which produces peculiar endosomes full of small intraluminal vesicles (ILVs) named multivesicular bodies (MVBs), and apoptotic bodies (Figure 1). These are a heterogeneous population of vesicles released from cells undergoing apoptosis, which contain not only organelles such as mitochondria and nuclear fragments, but also several bioactive molecules, including proteins and nucleic acids [7].

Exosomes are smaller in size (30–150 nm) as compared to microvesicles (100–1000 nm) and apoptotic bodies (50–500 nm) [8]. Nevertheless, the sizes of exosomes, microvesicles, and apoptotic bodies partially overlap, so the current physical methods based on size used for EV separation, namely differential centrifugation, cannot separate them. For this reason, it is currently considered more appropriate, when differential ultracentrifugation or other physical methods are used as separation procedures and cells are not undergoing significant apoptosis, to refer to medium/large EVs (m/lEVs), which sediment at lower speeds (10,000 *g* or 10 k fraction) and are enriched in microvesicles, or to small EVs (sEVs), which sediment at high speeds (100,000 *g* or 100 k fraction), and are enriched in exosomes [9,10].

To further complicate the picture, recent investigations have provided evidence that within sEVs and m/lEVs, further separation procedures such as density gradient centrifugation can separate different populations, i.e., low-density and high-density sEVs and low-density and high-density m/lEVs [11]. Besides, ultrastructural studies demonstrated that the same cells can release morphologically different EVs [12] and biochemical analysis of EV nucleic acids cargo showed that different vesicle subpopulations are characterized by a different content of nucleic acids [3]. These results have definitively established that most studies carried out in the past to elucidate the physiological effects of EVs on target cells and/or on their therapeutic potential referred to heterogeneous populations of vesicles due to the limits of the separation methods available. For this reason, part of the controversy with respect to the physiological and pathological function of EVs, as well as on therapeutic and diagnostic potential, may be a consequence of the enrichment of various EV subpopulations due to different separation methods. Our comprehension of EV biology is expected to improve in the future concomitantly with the development of better separation methods.

Despite these methodological obstacles, there is wide consensus that stress conditions, including lysosomal dysfunction due to undigested substrate accumulation, affect EV release. Lysosomal storage disorders (LSDs) are characterized by the accumulation of undigested substrates within the endosomal–lysosomal system, leading to endosomal/lysosomal dysfunction. Here we review studies investigating the role of lysosomal and autophagy dysfunction on the release of EVs, with a focus on studies investigating the release of EVs in LSD models of both genetic and pharmacological origin. The role of EV release in both alleviating intracellular storage of undigested materials and spreading the pathology to the neighbor cells has been also taken into account.

## 2. Extracellular Vesicle Biogenesis

### 2.1. Exosome Biogenesis

Although “pure” EV populations are difficult to separate, some aspects of exosome and microvesicle biogenesis have been clarified. Within the cell, vesicles are generated by inward budding of the late endosome (LE) membrane. The result of this process is a peculiar LE which becomes full of small vesicles in its lumen. For this reason, these vesicles are termed ILVs, and a LE full of them is an MVB. MVBs can either fuse with lysosomes to degrade their cargo or with the plasma membrane to release ILVs extracellularly. Once released in the extracellular environment upon MVB exocytosis, ILVs are known as exosomes. The biogenesis of ILVs may occur via different mechanisms, and more than one mechanism co-exists within the same cell [13]. In fact, the inhibition of a single pathway does not completely suppress the biogenesis and release of EVs, thus demonstrating that more than one mechanism is active at the same time within a specific cell type [14].

#### 2.1.1. Endosomal Sorting Complex Required for Transport (ESCRT)-Dependent Mechanisms

Exosome biogenesis can be classified into ESCRT-dependent and ESCRT-independent mechanisms. The ESCRT is a molecular machinery made up of several protein complexes and has a fundamental role in the degradation of the endosome ubiquitinated cargo via its sorting within the ILVs of MVBs. Subsequently, the cargo is degraded by MVB fusion with lysosomes. Currently, it has become evident that ESCRT machinery is also responsible for several cellular processes depending on membrane scission, such as plasma membrane division in cytokinesis and release of viral particles [15]. Therefore, it is difficult to dissect the role of the ESCRT proteins in exosome biogenesis without interfering with other important cellular processes.

ESCRT is composed of four protein complexes (termed ESCRT-0, ESCRT-I, ESCRT-II, ESCRT-III), a few associated proteins, namely Alix, and the ATPase VPS4, whose activity is responsible for ESCRT-III disassembly and neck scission [16]. The ESCRT-0 complex function is to select ILV cargo by binding to both endosomal membrane lipids and ubiquitinated proteins. The ESCRT-0 complex recruits the ESCRT-I complex, which includes the Tsg101 protein. This complex is also involved in cargo selection. ESCRT-I in turn recruits ESCRT-II, and both complexes control the initial formation of the inward endosomal membrane buds. ESCRT-II recruits the ESCRT-III complex, characterized by the presence of filaments consisting mainly of CHMP proteins 2, 3, and 4, which further induce the vesicles budding, until the ESCRT machinery is released by VPS4 ATPase [17].

While in some cell types Alix seems to be involved in the selection of exosome cargo [18], in cancer cells [19] Alix is involved in specific sorting of transmembrane protein to ILVs via protein–protein interaction. In fact, Alix interacts with the cytoplasmic protein syntenin, which in turn binds to the cytoplasmic tail of the plasma membrane protein syndecan, which is internalized during early endosome formation. Syndecan is a heparan sulphate proteoglycan that, through its glycan moiety, interacts with cell signaling factors in the extracellular matrix. The Alix–syntenin–syndecan axis participates, together with ESCRT I and III components, in the inward budding of ILVs, in a process that is also dependent on the small GTPase Arf6 and on phospholipase D2 (PLD2) [19,20].

#### 2.1.2. ESCRT-Independent Mechanisms

ESCRT-independent mechanisms in exosome formation became evident when it was reported that MVB biogenesis also took place in the absence of ESCRT proteins [21]. Lipid biophysical properties are important for membrane remodeling, and ESCRT-independent exosome biogenesis was shown to involve lipids. In a pivotal paper in 2008, Trajkovic et al. [22] showed that in mouse oligodendrocytes, exosome biogenesis was dependent on the generation of ceramide by neutral sphingomyelinase (nSMase). Ceramide is a cone-shaped lipid that may directly promote membrane budding. However, it also has signaling properties, so it could affect exosome release indirectly. In fact, it can be converted into sphingosine 1-phosphate (S1P), a metabolite with its own receptor on the MVB surface. In turn, S1P promotes the maturation of exosomes within MVBs [23]. Ceramide and S1P belong to so-called sphingolipid rheostat, a mechanism controlling cell fate via the equilibrium between these two molecules, as ceramide is anti-proliferative and pro-apoptotic, whereas S1P has pro-survival effects [24]. Besides, ceramide and S1P regulate autophagy [25,26], which is interconnected with MVB homeostasis. Therefore, the mechanism leading from nSMase inhibition to exosome release decrease may be more complex than a simple physical mechanism promoting membrane invagination, as initially thought.

An additional ESCRT-independent mechanism of exosome biogenesis relies on tetraspanins. They represent a family of membrane proteins with four transmembrane alpha-helices and include many proteins which are considered among the best markers for exosomes, such as CD63, CD9, and CD81, although several studies have provided evidence that there are currently no available markers that are exclusively present on exosomes [10]. Tetraspanin can organize the so-called tetraspanin-enriched domains (TEM), which are highly ordered membrane regions that can interact with many proteins involved in cell adhesion and signaling. These domains may favor membrane invagination, possibly interacting with actin polymerization [27]. The evidence of a tetraspanin-dependent mechanism was provided by studies demonstrating that specific proteins, such as the premelanosome protein (PMEL), are addressed to exosomes via CD63 [28,29] and that CD9 facilitates the loading of β-catenin into EVs [30].

### 2.2. Microvesicle Biogenesis

The generation of microvesicles has been far less investigated as compared to the generation of exosomes. The first step of microvesicle generation is the accumulation of cargos, possibly by interaction with specific lipids in plasma membrane domains, on the cytosolic side of plasma membrane. Microvesicle production requires the outward budding of cell membrane, and various mechanisms have been implicated in this event. It has been recognized that ESCRT machinery is relevant for the biogenesis of microvesicles. Specifically, Tsg101, a component of ESCRT-I complex, has been shown to be implicated in the budding of HIV particles, together with another important ESCRT component, the VPS4 ATPase [13,31]. There is also evidence that microvesicle biogenesis is mediated by calcium-dependent processes, as Ca^2+^ acts as a co-factor activating several targets involved in microvesicle biogenesis. Specifically, Ca^2+^ activates proteins involved in cytoskeleton remodeling by binding of actin to proteins such as gelsolin [32], a step which is necessary to promote outward budding. In addition, Ca^2+^ is an activator of several enzymes acting on membrane phospholipid to alter its asymmetry, such as flippase, floppase, and scramblase [33]. These enzymes catalyze the translocation of phospholipid across plasma membrane and are therefore involved in regulating plasma membrane asymmetry, another key step to control in order to promote outward membrane budding [34]. Additional factors involved in the regulation of plasma membrane-shedding vesicles are small GTPases of the Rho family, that also act on actin beneath plasma membrane, and the small GTPase Arf6, which is involved in vesicular trafficking [35].

## 3. EV Release

The release of exosomes and microvesicles presents different characteristics. In the case of exosomes, MVBs may function as a depot of exosomes, that may be released either constantly (constitutive secretion) or upon a specific stimulus (inducible secretion). In the case of microvesicles, no storage is possible and, upon their outward budding from the plasma membrane, they are promptly released. According to this behavior, it is evident that mechanisms inducing the biogenesis and the release of microvesicles are closely related, whereas these two moments can be temporally separated for exosomes. Therefore, it can be speculated that the two types of vesicles may have different extracellular signals to transmit.

ILVs formed within MVBs can either fuse with the lysosome to degrade their cargo or alternatively with the plasma membrane to release exosomes. The degradative function of ubiquitin tagged proteins via formation of exosomes is fundamental, as demonstrated by the evidence that mutations in CHMP2B, a key component of ESCRT-III, were identified in frontotemporal dementia (FTD) linked to chromosome-3 (FTD-3) and in amyotrophic lateral sclerosis (ALS), pathologies characterized by the accumulation of ubiquinated inclusion in neurons [36]. Therefore, it can be hypothesized that once molecules have been sorted into exosomes, they represent unwanted material destined to be eliminated, either by degradation or EV release. Nonetheless, the molecular mechanisms and signals underlying either the degradation into lysosomes or the extracellular release of vesicular cargo are not currently known. In addition, vesicles released extracellularly also have the capacity to transmit cell signals and therefore, mechanisms responsible for their release have a fundamental role in regulating signal transmission.

G-proteins such as the Ral small GTPase Ral1 and the small GTPases Rab27a and Rab27b [37,38] control the intracellular movement of MVBs towards fusion with the plasma membrane; then the fusion itself is controlled by one or several soluble N-ethylmaleimide-sensitive fusion protein attachment protein receptor (SNARE) complexes. Subsequently, the release of exosomes can be regulated by different triggers. As in the case of microvesicles, the intracellular Ca^2+^ level increase induces the release of exosomes, therefore any signaling pathway raising intracellular calcium concentration represents a potential extracellular trigger for exosome release. An increased membrane level of phosphatidic acid, generated by PLD2 or by diacylglycerol kinase (DGK), has been also associated with an increased release of exosomes [20,39]. In neurons and glial cells, the depolarization and neurotransmitter stimulation also represent a stimulus for exosome release [13]. From a genetic point of view, induction of p53 has been also shown to lead to increased release of exosomes [40]. Similarly, stress conditions that have an impact on p53 status, such as cellular senescence induced by irradiation and oncogene activation, have also been described to be associated with an increased release of exosomes [8,41,42]. Another well recognized cellular stress condition which is associated with an increased release of exosomes is hypoxia [43]. Extracellular signals acting on cell surface receptors can lead to an increased release of exosomes. In fact, it has been shown that activation of purinergic receptor by ATP is a stimulus for exosome release [44], as well as the action of heparanase, which regulates the syntencan/Alix pathway via syndecans [45]. Conversely, ISGylation, i.e., the addition of the ubiquitin-like protein interferon-stimulated gene (ISG)15 to different targets, has been demonstrated to decrease the release of exosomes [46].

In the case of microvesicles, their release is closely connected with their neogenesis and is controlled by contraction of actomyosin close to specific membrane microdomains [47], in the presence of small GTPases of the Rho family such as Rho, Rac, and Cdc42 [48]. The generation of spiral filaments by ESCRT-III complex and the membrane fission induced by the Vps4 ATPase are also relevant to obtain membrane fission and consequent release of vesicles [15]. Besides, some of the triggers that have been described as regulating exosome secretion have been shown to be involved also in promoting microvesicle biogenesis and release. Intracellularly, this has been demonstrated for Ca^2+^, which prompts the release of exosomes and can also stimulate the production of microvesicles [13]. Similar findings were reported for extracellular ATP- and ADP-activating purinergic receptors. Hypoxia conditions were also described to increase the production of microvesicles [49]. However, in view of the difficulties in separating exosome and microvesicle populations with currently available methods, whether specific stimuli increase the release of small EVs, medium/large EVs, or both, has not been elucidated, and often studies have investigated only a single type of vesicles. Therefore, it can be argued that as further studies focused on the release of different types of vesicles will be provided, the current picture would change.

## 4. Lysosomal Dysfunction and EVs

Lysosomes are acidic organelles surrounded by a single membrane, whose function is to degrade and recycle intracellular and extracellular components via autophagy or endocytosis, respectively. Although for decades they have been considered just degradative compartments, it later emerged that they represent a metabolic hub finely controlling the level of many metabolites. As for examples, lysosomes regulate amino acid levels tuning protein degradation according to cellular needs [50,51].

Age-related neurological disorders are characterized by the presence of undigested material within the endosomal/lysosomal compartment and are associated with autophagy/lysosomal dysfunction [52,53]. Investigation of these disorders reported that these alterations have an impact on EV release. In the case of Alzheimer’s disease (AD), the pathology is characterized by the intracellular accumulation of hyperphosphorylated tau protein aggregates and by the altered processing of amyloid precursor protein (APP) into amyloid β peptide (Aβ), which accumulates intracellularly and extracellularly into amyloid plaques [54]. Much evidence has associated this disorder to lysosomal dysfunction. It has been demonstrated that amyloid plaques, which are the hallmark of the pathology, are characterized by the co-localization of lysosomal enzymes [55], and in a peripheral model of Alzheimer’s disease lysosomal glycohydrolases are upregulated, whereas cathepsin D, a lysosomal protease which has been also implicated in APP processing, is decreased [56,57]. Besides, mutations involved in the familial form of several neurological diseases have been shown to affect lysosomal function [58,59,60].

Parkinson’s disease (PD) is characterized by the loss of dopaminergic neurons from the substantia nigra and the presence of intraneuronal inclusions called Lewy bodies, which are made up mostly of aggregated α-synuclein. In a minority of cases (5–10%) PD is genetically transmitted, with autosomal dominant or recessive inheritance [61]. In addition to mutation in α-synuclein, the pathology has been correlated to several additional mutations and many of them have been shown to alter lysosomal function [62]. As a matter of fact, homozygous mutations in the recessive genes Parkin, PINK1, and DJ1, account for most of the young-onset cases. These three proteins have different biochemical properties (Parkin is a ubiquitin ligase, Pink1 is a protein kinase, DJ-1 is involved in mitochondria homeostasis) but act in a common pathway that triggers the clearance of damaged mitochondria by lysosomes (mitophagy). This pathway is relevant as autophagic degradation is the only manner to get rid of impaired organelles and many investigations support a role for mitochondrial dysfunction in the development of sporadic PD [63,64]. Further, additional genes associated with PD in an autosomal dominant transmission are involved in lysosomal homeostasis [65]. Besides, a mutated *GBA* gene which encodes for the lysosomal enzyme β-glucocerebrosidase, whose deficiency causes Gaucher’s disease, is a major risk factor for PD [66]. Moreover, other neurological disorders have been associated with endo-lysosomal dysfunction, such as FTD, ALS, Huntington’s disease (HD) and hereditary spastic paraplegia (HSP), leading to the hypothesis that dysfunction of lysosomal trafficking and signaling are converging mechanisms in the development of neurological disorders [53,67].

The main pathological feature of AD and PD is the accumulation of toxic protein aggregates. Early investigations rapidly demonstrated that these aggregates can be released outside the cell via EVs, thus suggesting that EVs may represent an additional manner to discard toxic material when intracellular digestion does not work properly, because of an impairment of the autophagic flux and/or lysosomal degradation. Evidence has been provided that pathological cell models release EVs containing molecules that are the main pathological hallmarks of either AD, i.e., Aβ peptide [68], APP fragments [69,70] and Tau [71], or PD, i.e., α-synuclein [72]. The presence of pathological proteins in EVs has been described also for other neurological disorders [73]. For example, ALS has been shown to be associated with mutations in two genes, i.e. superoxide dismutase and TAR DNA-binding protein (*TARDBP)* and both these proteins have been found in EVs [74,75,76]. In the case of HD, which is associated with the trinucleotide (CAG) repeat expansions of the Huntingtin gene, it has been reported that the protein can be transmitted via EVs, although other mechanisms of secretion have been also proposed [77,78].

The endosomal/lysosomal system is made up of intracellular membranous compartments that are dynamically interconverted and undergo morphological and biological changes during their maturation. The early endosome receives macromolecules extracellularly from the plasma membrane via endocytosis and intracellularly from the Golgi. These substances may be recycled back to the cell surface through recycling endosomes, to the trans-Golgi network by retrograde transport, or instead proceed towards LEs. LEs receive also material from the autophagic pathway, then integrate outgoing traffic to the lysosomes for degradation and the plasma membrane for secretion. As already mentioned, a subset of LEs is subjected to the inward budding of their membrane, originating MVBs full of ILVs, that can either fuse with the lysosome for degradation or with the plasma membrane to release vesicles extracellularly. Due to this interconnection, it has been argued that lysosomal dysfunction must have an impact on EV biogenesis and release. In fact, the presence of pathological toxic proteins which are hallmarks of neurological diseases into EVs was shown to be reinforced when lysosomal function was inhibited using agents able to raise the lysosomal pH. Treatment with bafilomycin A, a V-ATPase inhibitor that blocks the autophagic flux by preventing autolysosome acidification, increases the level of α-synuclein secreted in EVs [79,80]. Similarly, chloroquine increases the release of the C-terminal fragment of APP protein in EVs [81] and the amount of Aβ secreted in association with EVs [82].

Further evidence of a link between EVs release and endosomal-lysosomal system status comes from the finding that perturbation of autophagic process affects EV release, even if the field is far from being satisfactorily elucidated [83]. A few studies have provided evidence that autophagy key genes, such as Atg5 and the Atg12–Atg3 complex, are also involved in modulating EV release. In fact, exosome production is strongly reduced in cells lacking Atg5 and Atg16L1, but this is independent of Atg7. Atg5 disassociates the V_1_V_0_ -ATPase [84]. As in the case of pharmacological agents raising endosomal-lysosomal pH, i.e., the above mentioned bafilomycin A and chloroquine, the dissociation of the V_1_V_0_-ATPase raises acidic organelle pH and this in turn stimulates the fusion of MVBs with plasma membrane and the extracellular release of their ILVs [84]. The Atg12–Atg3 complex, which is required in early steps of autophagy, interacts with Alix, a protein that has a crucial importance for the function of ESCRT complex, as it participates in EV biogenesis by at least two different ESCRT-dependent mechanisms [85].

Autophagy can be modulated not only genetically but also pharmacologically, and pharmacological modulation has been shown to have an impact on EV release. Conditions inducing autophagy, such as starvation or rapamycin treatment, decrease exosome release [86]. On the other hand, autophagy inhibition using wortmannin prompted exosome release [87]. Besides, preventing autophagosome–lysosome fusion with bafilomycin A increases exosome release [46,88]. This evidence is in agreement with what we have already mentioned above, i.e., Alvarez-Erviti et al. [79] and Vingtdeux et al. [81] observed an increase of pathological protein aggregates in exosomes of AD and PD cell models following treatment with bafilomycin A and chloroquine, respectively.

It is worth reminding that the transient alleviation of toxic protein accumulation in neurons via EV release when the autophagic flux is impaired may represent a double edge sword, as EVs containing pathological misfolded proteins can be taken up by surrounding cells, thus contributing to the pathology transmission. In other terms, what is fit for a cell is not necessarily fit for the overall tissue and/or organ. Furthermore, it must be considered that some studies showing the release of toxic protein aggregates via EVs must be analyzed carefully, as this release could also occur via secretion of autophagosomes, which are-co-separated with exosomes and/or microvesicles, via the so-called “secretory autophagy” pathway, and not by EVs. In fact, recent studies have also demonstrated that a few proteins, such as interleukin (IL)1β, are released via unconventional secretion of autophagosomes [89]. However, previous studies had initially identified IL1β as released with microvesicles [90] and exosomes [91]. Therefore, vesicular structures released extracellularly and containing pathogenic proteins can be more heterogeneous than previously thought and further investigations are needed to unravel the relation between unconventional secretory processes and EV release. Another issue that has been poorly investigated is the effect of bafilomycin A or chloroquine treatment on EV uptake. In fact, in many studies, authors did not specifically distinguish between an increase of the EV release and a decrease of the EV re-uptake. However, long-term treatment with bafilomycin has been previously established to impact receptor mediated endocytosis and protein secretion [92,93].

## 5. Lysosomal Storage Disorders and EVs

The finding that dysfunction of the autophagic-lysosomal system affects EV release implicates that intra or extra-cellular stimuli perturbing lysosomal function and/or autophagy, namely the presence of undigested substrate within the endo-lysosomal compartment, may have an impact on EV release. Lysosomal storage disorders (LSDs) are a wide group of pathologies characterized by the intralysosomal accumulation of substrates, leading to lysosomal dysfunction, and these features have been often compared to age-related neurological disorders [94]. LSDs are inherited disorders caused by mutations in genes encoding lysosomal proteins. About 50 of them arise from mutations in lysosomal hydrolases, but mutations in integral membrane proteins, lipid and ion transporters or regulator proteins have also been reported [95]. The deficit of the mutated protein leads to the gradual accumulation of undigested or partially digested material into lysosomes (“primary storage”) and lysosomal dysfunction, which culminates with severe cellular damage and cell death. The composition of accumulated materials depends on the nature of the primary genetic defect and is characteristic for each LSDs. Moreover, in many cases, a “secondary storage” of undigested material can also occur as result of other endo-lysosomal pathway alterations derived from the main defect. Therefore, the pathogenic cascade leading to cell dysfunction and death is complex and currently not fully understood.

Because of the complexity of the pathophysiology of these disorders and the different organs and tissues involved in each case, LSDs are clinically heterogeneous. However, some common clinical manifestations are present, i.e., hepatosplenomegaly, bone abnormalities, cardio-respiratory problems, and developmental delay. Moreover, most of LSDs present neurological deficits and neurodegeneration [95,96]. Up to today, only few LSDs are treatable and most of them, namely those with neurological implication, can only be managed symptomatically and by supportive care. The most common therapeutic strategy currently approved is enzyme replacement therapy (ERT) [97,98], but substrate reduction therapy (SRT), pharmacological chaperone therapy (PCT), and hematopoietic stem cell therapy (HSCT) are also applied in some cases [99,100,101,102,103]. A combination of ERT, SRT, and PCT is currently available for Gaucher disease, Fabry disease, and a few mucopolysaccharidoses (MPSs) [104]. For other pathologies with neurological involvement, such as metachromatic leukodystrophy, gene therapy protocols are being developed [105]. In other cases, characterized by neurological symptoms, such the Tay–Sachs and Sandhoff gangliosidoses, no effective treatment beyond palliative care is possible [106]. Nevertheless, considerable progress has been made in the last decades in the field of LSD research and an increasing number of various therapeutic protocols are now undergoing clinical trials or preclinical development [95,107,108,109,110].

Given the involvement of lysosomes in several processes responsible for the maintenance of cell homeostasis, alteration of their function gives rise to dysregulation of fundamental cellular pathways, including not only intracellular trafficking and autophagy, but also Ca^2+^ homeostasis and oxidative stress response [111,112]. In pathological models of LSDs, stored material has been shown to be released outside the cell by lysosomal exocytosis, i.e., by fusion of lysosomes with the plasma membrane [113]. A first evidence comes from a study on metachromatic leukodystrophy (MLD), an LSD caused by mutations in the lysosomal hydrolase arylsulfatase A (ASA) [114]. The enzymatic deficit leads to the storage of sulfatides mainly in white matter, bile ducts, and distal tubule kidney cells. The study showed that Ca^2+^ induced lysosomal exocytosis in cultured primary kidney cells of deficient mice resulted in delivery of storage material to the extracellular environment, indicating that cells can eliminate the storage material by exocytosis and giving a possible explanation for the presence of sulfatides in body fluids of MLD patients.

**Figure 2:**
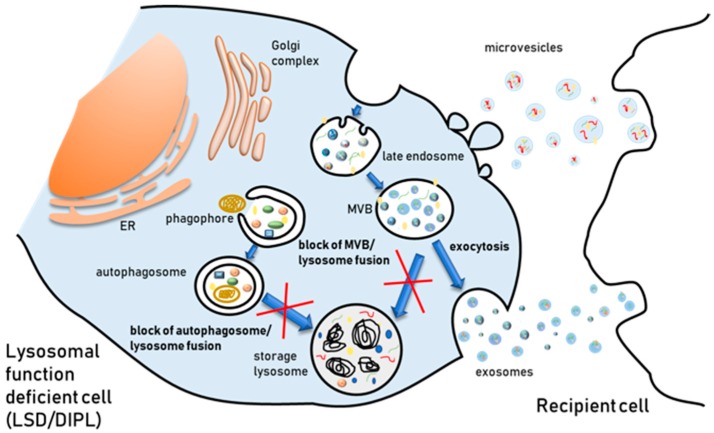
Representation of EV release induced by lysosomal storage in LSDs. Lysosomal storage results from the presence of undigested substrates caused by the accumulation of material either due to genetic deficiency of lysosomal enzymes or to treatment with drugs inducing the accumulation of phospholipids.

More recently, it has been reported that in LSDs the release of undigested substrates may occur also via EVs (Figure 2). In fact, an increase of exosomal cholesterol secretion has been observed in fibroblasts derived from patients affected by Niemann–Pick type C1 (NPC1) disease [115]. NPC is characterized by the abnormal intralysosomal accumulation of cholesterol and sphingolipids due to mutations in the *NPC1* gene, encoding for an intracellular cholesterol transporter localized in the lysosome membrane. A similar increase of cholesterol exosomal release was also observed by threating oligodendroglial cells with cholesterol or U18666A, which are well known inducers of intracellular cholesterol accumulation in LEs/lysosomes [115]. Authors suggested that the exosomal release of cholesterol may represent a cellular response to both minimize damage caused by the storage and maintain cellular cholesterol homeostasis. Another study which aimed to investigate the alterations of autophagic/mitophagic pathway in a lymphocyte cell model of Nieman–Pick type B (NPB) revealed a more robust and abundant exocytosis processes in pathological cells with respect to normal cells [116]. NPB is characterized by the pathological accumulation of sphingomyelin within lysosomes caused by mutations in the *SMPD1* gene encoding acid sphingomyelinase. The analysis of the composition of vesicles released in the extracellular environment of NPB B lymphocytes showed the presence of microvesicles and lipid particles. Authors suggested that the release of lipid particles, which was also associated with lysosomal exocytosis, may allow cells to overcome, at least in part, the blockage of the traffic resulting from the intralysosomal lipid accumulation, contributing to maintain cellular lipid homeostasis [116]. Again, this finding indicates that the release of EVs could play a protective role by providing an alternative route to dispose waste when lysosomes are overloaded.

Although it has not become clear whether the intracellular storage of undigested materials itself has beneficial/adverse effects, the evidence that intracellular accumulation of undigested substrates can be alleviated by lysosomal exocytosis and EV release, implicates that the stimulation of these processes may represent a useful therapeutic strategy to remove the storage material from lysosomes, possibly alleviating the pathology. For instance, treatment with β-cyclodextrin, a naturally occurring cyclic oligosaccharide able to reduce lipid accumulation by promoting lysosomal exocytosis in a Ca^2+^-dependent manner [117], has proved to be effective to reduce the level of storage in brain and several peripheral organs of different animal models of NPC [118,119]. The use of cyclodextrin derivatives for the treatment of NPC patients is currently under investigation in late stage clinical trials [120].

Lysosomal exocytosis is under the control of the transcription factor EB (TFEB) [121], a master gene regulating lysosome biogenesis and autophagy [122,123]. TFEB activates lysosomal exocytosis by raising intracellular Ca^2+^ in a mucolipin-1-dependent manner [121]. Therefore, it has been speculated that promoting TFEB activation could be an effective strategy for removing storage vesicles from overloaded lysosomes. Medina et al. demonstrated that TFEB overexpression induced lysosomal exocytosis and promoted cellular clearance in cell models of various LSDs, including multiple sulfatase deficiency (MSD) and mucopolysaccharidoses type IIIA (MPS-IIIA), both characterized by severe neurodegeneration due to glycosaminoglycan (GAG) accumulation, as well as Batten disease, a type of neuronal ceroid lipofuscinosis, and Pompe disease, a type of glycogenosis [121]. In this study, a significant decrease of stored material was observed in all LSDs tested. Moreover, an increase of secreted GAG in MSD and MPS-IIIA was reported, indicating that cellular clearance was achieved by exocytosis. In the same study, the effect of TFEB overexpression was also tested on a mouse model of MSD. TFEB overexpression promoted not only a decrease of stored GAG but also the reduction of associated pathological manifestations such as inflammation and cell death [109]. Based on this finding, pharmacological or genetic TFEB activation has been proposed as a feasible therapeutic approach to promote cellular clearance for those disorders, such as LSDs and neurodegenerative diseases, characterized by lysosomal dysfunction or overload [121,124]. Although the stimulation of lysosomal exocytosis has been proved as a useful strategy to prompt cell clearance, at least in vitro and in animal models, it is not actually clear whether the induction of EV release might provide an additional route to remove intracellular storage and ameliorate cell homeostasis.

However, the effect of increased extracellular “waste” on neighboring cells should be carefully evaluated. For instance, recent finding indicates that sphingolipid cargo of EVs may be responsible of detrimental effect on the recipient cells [24] and preliminary studies have considered the potential neurotoxic effect of sphingolipids released with EVs in pathological conditions, such as sphingolipidoses, a group of LSDs characterized by the accumulation of specific sphingolipids, depending on the enzymatic defect [125]. EVs enriched with pathogenic material may be taken up by neighboring cells, possibly contributing to the spreading of the disease, but it is currently unknown how this mechanism works and many aspects related to EV uptake and processing in recipient cells still need to be elucidated to address this issue. Pituch et al. observed an increase on exosomal shedding of platelet-derived growth factor A (PDGFRα), a molecule controlling the proliferation and survival of oligodendrocyte progenitor cells, in MLD multipotential neural precursor cells [126]. These cells are deficient in ASA activity and accumulate sulfatides, leading to a progressive destruction of white matter throughout the nervous system. Authors suggested that sulfatides may play a role in exosomal biogenesis, and increased sulfatides levels associated with MLD may be responsible for the increased shedding of PDGFRα. This in turn reduces the production of oligodendrocyte progenitor cells and oligodendrocytes from multipotential neural precursor, impairing myelin synthesis [126]. Therefore, sulfatide-loaded EVs released by ASA-deficient cells may behave as a shuttle system that transfer toxic lipids to neighboring cells disseminating their pathogenic effect. Krabbe’s disease, also known as globoid cell leukodystrophy, is a neurodegenerative glycosphingolipidosis caused by the deficiency of the lysosomal enzyme galactosyl-ceramidase. It is characterized by the accumulation of undegraded galacto-lipids, particularly psychosine. It has been reported that the high level of psychosine in myelin membranes from Twitcher mice, an animal model of Krabbe’s disease, alters myelin fluidity, leading to increased membrane rigidity and loss of myelin by fragmentation of the membrane through microvesiculation [127]. This finding suggests that psychosine may play a direct role on the pathophysiology of Krabbe’s disease contributing to demyelination. Consequently, EVs derived from fragmentation of psychosine-enriched membranes may propagate damage of myelin structure by transferring the pathogenic glycosphingolipids and causing structural changes to neighboring cell membrane.

The rationale of stem cell therapy assumes that donor cells may both (1) transdifferentiate and integrate into host tissues and (2) deliver their content to affected cells, reversing the pathogenic condition. There is evidence indicating that EVs secreted by transplanted cells may effectively contribute to the efficacy of stem cell therapy [128]. In the last decades, many LSDs have been treated with transplantation of stem cells derived from different source resulting in clinical improvement [129,130]. However, the molecular mechanisms by which wildtype enzyme is transferred to mutant cell is not been fully investigated in most cases. EVs released by normal cells could be taken up by cells affected by a mutation causing LSDs, thus representing a source to supply the missing protein to deficient cells. For instance, Iglesias and co-workers demonstrated that mesenchymal stem cells (MSCs) were able to reduce the pathologic accumulation of cystinotic cells in vitro by a paracrine effect [131]. Cystinosis is a rare LSD characterized by massive accumulation of cystine in several body tissues caused by mutations of the *CTNS* gene, which encodes for cystinosin, a cystine-selective transport channel localized in the lysosomal membrane. In one study, MSCs derived from amniotic fluid or bone marrow were shown to release into the culture medium microvesicles containing wild type cystinosin protein and CTNS mRNA, which were up-taken by co-cultured CTNS-deficient fibroblasts or proximal tubule cells, reversing the pathologic cystine accumulation [131]. Of note, this study shows that vesicles released by stem cells may deliver to neighboring cells not only soluble material but also membrane-bound proteins such as cystinosin, which can be transferred to the lysosomal compartment by membrane fusion events and correct the defect. Another study that aimed to explore alternative treatments for corneal defects in MPS VII patients demonstrated the existence of intercellular trafficking between donor human umbilical mesenchymal stem cells (UMSCs) and host cells [128]. A co-culture experiment with intracellular labeled UMSCs and primary MPS VII skin fibroblasts isolated from the mouse model of the disease showed that UMSCs secreted in the culture medium vesicles were taken up by deficient cells and fused with lysosomes, delivering active β-glucuronidase in host cells.

Therefore, EVs released by transplanted stem cells may indeed contribute to the therapeutic efficacy of cell therapy for congenital metabolic disorders such as LSDs. This is of particular interest because small EVs have shown the ability to cross the blood–brain barrier and could therefore represent a therapeutic vehicle for disorders showing accumulation within the central nervous system.

## 6. Pharmacological Models of Lysosomal Storage and EV Release

The accumulation of specific molecules in the endosomal/lysosomal system can be induced by pharmacological agents. This condition shares morphological and pathological hallmark features with several LSDs. An example comes from drug-induced phospholipidosis (DIPL), a condition characterized by an intracellular accumulation of phospholipids as lamellar bodies inside lysosomes [132]. Several drugs can induce DIPL and most of these belong to the cationic amphiphilic drug (CAD) family [133]. CADs are amphiphilic molecules sharing a similar chemical structure, i.e., a hydrophobic ring on the molecule and a hydrophilic chain with an amine group [134]. The hydrophobic portion of these molecules gives them the ability to easily cross biological membranes, whereas the presence of the weakly basic amine group drives their accumulation within acidic subcellular compartments such as lysosomes. Once inside the acidic lumen of the organelles their weak base moieties become protonated and the ionized forms of CADs are not able to diffuse out [135]. The trapped CADs alter lysosomal functions and cause the accumulation of various lipid classes in the lumen of the organelle. In fact, CADs are often used in cell culture models to induce a LSD phenotype [136,137].

The wide therapeutic spectrum of CADs and the multiplicity of organs/tissues accumulating phospholipids complicate the identification of the molecular mechanisms underlying DIPL [123], even if the common event underlying the various DIPL models is the protonation of the drug within the lysosomes, whose first consequence is lysosomal luminal alkalization which, in turn, causes the impairment of lysosomal function [138,139]. It has been reported that several PL-inducing CADs (e.g., amiodarone, chlorpromazine, chloroquine, gentamicin) inhibit the activity of phospholipases A1, A2, and C [140]. It is not clear whether CADs form CAD/phospholipid complexes resistant to phospholipase activity or directly inhibit the enzymes [140]. Other in vitro and in vivo studies demonstrate that CADs also influence the biosynthesis of phospholipids and modify the expression of several genes related to lysosomal functions [134]. In line with this evidence, Ikeda and coworkers [135] demonstrated an increase of lysosomal enzyme activity in cell culture medium in association with DIPL induction, possibly caused by a blockade of lysosomal enzymes targeting via the mannose phosphate receptor. Therefore, the determination of the specific activity of lysosomal enzymes released into cell culture medium was proposed by authors as a relatively simple screening method to predict DIPL induction, and its validity as screening method was later confirmed by other studies [141,142].

A few investigations have provided evidence that several CADs are able to affect autophagy in cell lines [143,144]. However, this finding must be carefully examined, as many CADs do not induce an increase of autophagic degradation but only an accumulation of autophagic markers such as LC3 protein, due to the block of autophagosome-lysosome fusion and LC3 recycling caused by lysosomal alkalinization. In fact, Morisette and coworkers [145] have shown that the autophagic degradation of the intracellular vacuoles formed by the accumulation of CAD–phospholipid complexes is inhibited by the alkalinization of the lumen of lysosomes driven by CADs. Thus, cells evolve towards a persistent but inactive autophagy maintained by the continuous influx of drugs. More recently, it has been demonstrated that amiodarone-induced phospholipidosis activates TFEB in hepatocytes [142], but this activation is not enough to increase autophagic flux and prevent phospholipid accumulation, although TFEB overexpression prior to amiodarone treatment alleviates phospholipid accumulation.

Since the trafficking of MVBs depends on the lysosomal status, it is conceivable that the inhibition of the lysosomal function and/or the overloading of undegraded materials within endosomes/lysosomes in DIPL could influence EV biogenesis in MVBs. DIPL share morphological and pathological hallmark features with LSDs and the trapping of lipids within lysosomes is a common event associated with DIPL and LSDs. For example, similar pathological and morphological features have been reported for Fabry’s disease and DIPL induced by chloroquine [146]. In fact, this antimalaria drug is a CAD and PL-inducer, commonly used at low concentrations to raise lysosomal pH and block autophagic flux by preventing autophagosome–lysosome fusion [147]. It is also able at high concentrations to induce phospholipid accumulation, mostly in kidneys [148]. In Fabry’s disease, the deficiency of α-galactosidase A causes accumulation of globotriaosylceramide. Analysis of patients with Fabry’s disease or subjects treated with suitable concentrations of chloroquine show the presence in both cases of inclusion bodies in specific tissues, i.e., renal, endothelial, and striated myocardial muscle cells [146]. Besides, several CADs have been shown to induce non-esterified cholesterol accumulation in lysosomes [149,150,151] and, as previously mentioned, U18666A has been used to mimic many of the features of cells from subjects affected by different LSDs including NPC [152].

As for LSDs, the release of EVs in DIPL could also occur to alleviate lipid accumulation and counteract lysosomal dysfunction caused by lipid overload. So far, it is not clear if the involvement of the EVs in the removal of intracellular lipid overload induced by CADs is a common event associated with DIPL. Piccoli and coworkers [153] demonstrated that amiodarone, a well-known DIPL-inducing CAD, induces the formation of vacuoles and inclusion bodies in Baby Hamster Kidney (BHK) and Vero cells by interfering with late compartments of the endocytic pathway. Despite the similarities between cellular changes induced by amiodarone and NPC phenotype, differences in several mechanisms have been observed. In amiodarone-induced DIPL the impairment of late endosomal activities and the accumulation of undegraded materials do not modify the release of EVs. Further, the removal of cholesterol by methyl-β-cyclodextrin reverts the phenotype of cells treated with the NPC-inducer U18666A but does not decrease the number and the diameter of inclusion bodies in amiodarone-treated cells [153]. These results demonstrated that the impairment of late-endolysosomal compartment represents the crucial event leading to changes observed in amiodarone-treated cells and in NPC phenotypes. In any case, the type of lipid accumulation able to modify the release of EVs and the precise exocytic pathways activated by cells to remove lipid deposits must be elucidated. More recently, Canfrán-Duque et al. [154] demonstrated that curcumin, which has been identified as a novel mechanistic target of rapamycin (mTOR)-independent activator of TFEB [155], partially reverses the U18666A-induced endosomal cholesterol accumulation by conveying cholesterol out of the cell via EV secretion. In line with this, it has been also demonstrated that curcumin promotes cholesterol clearance from antipsychotic-treated cells by stimulating the release of exosomes, thus contributing to mitigating the lipid deposition phenotype [156].

## 7. Conclusions

A large body of evidence suggests that under endosomal/lysosomal undigested material accumulation, cells package these potentially toxic molecules in vesicles that are dismissed in the extracellular environment. In LSDs, an important concern is that the investigations carried out so far do not give an understanding of whether the amount and molecular content of EVs is dependent on the mutated gene and, therefore, on the type of accumulated substrate (“primary storage”). LSDs characterized by lipid accumulation and DIPL have similar features, as in both cases they are characterized by the intracellular accumulation of lipids. Hence, it is conceivable to hypothesize that during DIPL the release and content of EVs may be altered. Although much key information on EVs isolated from cell models characterized by lysosomal dysfunction, such as LSDs and DIPL, is still missing, it can be foreseen that this area of investigation will contribute to elucidate not only the role of EVs in maintaining cell homeostasis by alleviating intracellular storage, but also their impact on the extracellular transmission of pathological signals contributing to spread of the pathology to the neighboring tissue.
